# Facilitating early diagnosis of lung cancer amongst primary care patients: The views of GPs

**DOI:** 10.1111/ecc.12704

**Published:** 2017-05-11

**Authors:** R. Wagland, L. Brindle, E. James, M. Moore, A.I. Esqueda, J. Corner

**Affiliations:** ^1^ Faculty of Health Sciences University of Southampton Southampton UK; ^2^ Primary Care and Population Sciences University of Southampton Southampton UK; ^3^ Executive Office The Nottingham University University Park Nottingham UK

**Keywords:** early diagnosis, general practice, lung cancer, symptom awareness, symptoms

## Abstract

Early diagnosis of lung cancer (LC) is a policy priority. However, symptoms are vague, associated with other morbidities, and frequently unrecognised by both patients and general practitioners (GPs). This qualitative study, part of a larger mixed methods study, explored GP views regarding the potential for early diagnosis of LC within primary care. Five focus group discussions (FGDs) were conducted with GPs (*n* = 16) at primary care practices (*n* = 5) across four counties in south England. FGDs were audio‐recorded, transcribed verbatim and analysed using a framework approach. Four broad themes emerged: patients’ reporting of symptoms; GP response to symptoms; investigating LC, and; potential initiatives for early diagnosis. GPs reported they often required high levels of suspicion to refer patients on to specialist respiratory consultations, and concerns of ‘system overload’ were prevalent. Greater access to more sensitive diagnostic investigations such as computed tomography, was argued for by some, particularly for symptomatic patients with negative chest X‐rays. GPs challenged current approaches to promoting earlier diagnosis through national symptom awareness campaigns, arguing instead that interventions targeted at high‐risk individuals might be more effective without burdening services already under pressure. Further work is needed to identify primary care patients who might most benefit from such targeted interventions.

## 1 Introduction

Lung cancer is the second most common cancer in the UK, with 45,500 new diagnoses per year (Cancer Research UK, 2016), and two‐thirds only diagnosed at late stage when curative options are limited (Hamilton, Peters, Round, & Sharp, 2005). One and 5‐year survival rates compare unfavourably with other European countries (Berrino et al., 2007; Coleman et al., 2011; De Angelis et al., 2014), and evidence suggests this disparity may be partly influenced by the UK's structure of primary care provision (Rose et al., 2015). Symptoms associated with lung cancer are also manifestations of other common comorbidities amongst smokers/ex‐smokers (NICE, 2015), making diagnosis difficult (Bowen & Rayner, 2002). The “symptom signature” of lung cancer is “harder to suspect” than other tumour sites for general practitioners (GPs), with 30% of patients subsequently diagnosed with lung cancer undergoing multiple (≥3) consultations prior to diagnosis (Lyratzopoulos, Wardle, & Rubin, 2014). GPs typically encounter around one new presentation of lung cancer every 8 months, giving relatively little experience in diagnosis (Hamilton et al., 2005).

Recent initiatives have sought to improve early diagnosis of lung cancer. For example, the national “Be Clear on Cancer: 3‐week cough” campaign was conducted in England between April and June 2012, with a reported significant increase in diagnoses on the same period in the previous year (Ironmonger et al., 2015). This increased diagnosis rate was accompanied, however, by corresponding increases in additional workload, over which GPs had little control (PULSE 2014).

To promote earlier diagnosis of lung cancer without overloading services already under pressure, we need to explore ways to shorten the intervals between the patient first noticing and appraising potential symptoms as requiring clinical attention and then seeking help from their GP, while also promoting the symptoms most favourable for GPs to investigate appropriately. This study was part of a mixed methods investigation of symptomatology and help‐seeking behaviour among primary care patients at high‐risk (≥50 years old, recent smoking history) (Wagland et al., 2016). We found a high prevalence among participants of both symptoms associated with lung cancer and comorbidities that manifested similar symptoms. Almost half of the participants reported symptoms associated with lung cancer in a questionnaire for whom we found from a clinical notes review did not consult their GP. We also identified a small, clinically relevant group of patients (*n* = 61/908, 6.7%) who reported experiencing symptoms associated with lung cancer, but whom we found had not consulted their GP for ≥12 months (Wagland et al., 2016). The aim of the qualitative element of the study reported here was to explore the views of GPs regarding how best general practice might facilitate timely diagnosis of lung cancer.

## 2 Methods

### 2.1 Setting and sample

Focus group discussions (FGDs) were used for data collection as they explore collective rather than individual experiences and reveal the nature and variety of participants’ views (Krueger & Casey, 2000). Participants were drawn from five primary care practices across four counties within southern England. Recruitment of practices was facilitated by close collaboration with the Primary Care Research Network, to ensure a representative range of practice size and social deprivation. FGDs took place between April and August 2014 within the practice premises of collaborating GPs, and lasted 1–2 hr (mean: 1 hr 25 min). An experienced qualitative researcher (RW) conducted the focus groups as moderator, with a second researcher (AI‐E) acting as observer and note‐taker.

### 2.2 Materials

An interview topic guide was developed to elicit participants’ views regarding specific symptoms, symptom combinations, severity and chronicity of symptoms reported by patients that would raise suspicions of lung cancer. GPs gave their views regarding the importance of nine symptoms experienced by patients subsequently diagnosed with lung cancer, which comprise the IPCARD (**I**dentifying Symptom **P**redictors of **C**hest **a**nd **R**espiratory **D**isease) questionnaire (Brindle et al., 2015). IPCARD, developed by members of the research team (Brindle et al., 2015), asks individuals about the following symptoms in lay terms to facilitate elicitation: tiredness; breathing changes; chest and upper body aches; cough; coughing up blood; non‐menopausal sweats; ongoing voice changes; unintentional weight loss; and noticeably more chest infections over a 12‐month period. GPs were informed of the preliminary findings from the survey and clinical notes review from the wider study (Wagland et al., 2016). Their views regarding perceived barriers and facilitators to early diagnosis were then explored and practical considerations of administering interventions to encourage primary care patients to consult for symptoms potentially indicative of lung cancer.

### 2.3 Analysis

Focus group discussions were transcribed verbatim and analysed using the computer programme Nvivo10 for assistance in structuring a framework analysis (Ritchie & Lewis, 2003). Framework analysis aims at facilitating applied research and its requirements to meet specific information needs and actionable outcomes and is conducted through a series of stages. The focus group moderator led the analysis, ensuring deep familiarisation with the data.

To facilitate data analysis, three researchers (RW, EJ, AIE) independently analysed one FGD and discussed findings with the full research team to agree upon an index of emerging themes. Thereafter, regular 2‐weekly discussions took place between the three researchers to review the development of a thematic framework and ensure analytical rigour. A thematic framework was developed, initially drawn from the topic guide, to identify the key concepts central to the symptomology associated with identifying patients with lung cancer, judging symptom severity and the potential for early diagnosis. During the analysis, other categories were derived from the data, including critiques of national symptom awareness campaigns; issues related to investigating lung cancer and fatalistic attitudes amongst some GPs.

The whole data set was then indexed according to these categories (indexing) and comparisons made both within and between them according to their thematic content, and data within categories summarised (charting). Relationships and associations between the categories were then identified (mapping and interpretation) that explained GPs’ views regarding the facilitation of timely diagnosis of lung cancer during the diagnostic interval.

### 2.4 Ethical approval

Ethical approval for the study was secured by the National Research Ethics Service (NRES) Committee South Central‐Southampton A on 20/05/2012 (12/SC/0049).

## 3 Results

In total, 16 GPs and two practice nurses participated in the five FGDs, and each comprised between three and five participants (Table 1). Participant GPs had a range of between 4 and 31 years in general practice (mean: 18.5 years). Practice sizes ranged between 3,430 and 11,670 (mean: 7,486), and represented areas of both higher and lower social deprivation (range of Index of Multiple Deprivation scores: 24.4–10.0).

**Table 1:** Focus group participants: gender and years in practice

Focus group	Practice size	Index of Social deprivation	Participants		
			Gender	Occupation	Years in practice
FG1 (*n* = 5)	7,870	19.1	Male	GP	4
			Male	GP	6
			Female	GP	22
			Male	GP	25
			Female	Practice Nurse	12
FG2 (*n* = 3)	6,400	10.0	Male	GP	18
			Female	GP	26
			Male	GP	31
FG3 (*n* = 3)	11,670	24.4	Male	GP	19
			Male	GP	26
			Male	GP	30
FG4 (*n* = 4)	8,060	12.8	Male	GP	4
			Male	GP	16
			Female	GP	25
			Male	GP	30
FG5 (*n* = 3)	3,430	15.1	Male	GP	6
			Male	GP	9
			Female	Practice Nurse	8
Total/mean	7,486				297/18.5

Four broad themes emerged from the data: patients’ reporting of symptoms; GPs response to specific symptoms; issues related to investigating suspected lung cancer, and; issues related to the potential effectiveness of early diagnosis interventions (Table 2).

**Table 2:** Thematic coding framework

Theme	Category
Patients reporting of symptoms	Subjectivity of patient symptom experience
	Patient symptom “stories” change between consultations
	Difficulty eliciting symptoms
	Patients do not always recognise symptoms
	Patients do not always report symptoms
	Patients often perceive symptoms as normal
GP response to symptoms	Identifying “alarm” Symptoms for lung cancer
	Importance of GP hunch/gut instinct
	Difficulty judging severity of symptoms experienced
	Previous non‐attendance at GP practice as an “alarm” symptom
Investigating for lung cancer	Low threshold of suspicion for ordering chest X‐rays
	Chest X‐ray as a “blunt instrument”
	High threshold of suspicion required for onward referral
	Need for greater diagnostic tools (eg, CT scanning)
Potential for early diagnosis	Fatalist attitude amongst GPs
	Critique of national symptom awareness campaigns
	Preference for practice‐led targeted interventions over national awareness campaigns
	Preference for interventions targeting patient types rather than particular symptoms

### 3.1 Patients’ reporting of symptoms

The subjective nature of symptom experience made appraising the severity and seriousness of symptoms reported by patients difficult for GPs, especially for symptoms such as fatigue and chest pain for which limited objective measurements exist. GPs described how many older patients with smoking histories had several comorbidities, and as the extract below illustrates, GPs recognised that consequently those patients often believed it was normal to experience symptoms such as cough, fatigue and breathlessness, and consequently did not report them even when they worsened.

GP1: Until you ask the question, [patients] change their boundaries of what they do, to accommodate how they feel. They're not actually aware sometimes that their shortness of breath has got worse, they just don't walk so far, or they don't do this or that, and until you actually ask the question, they wouldn't come and see you because they're not aware of it.

GP2: They didn't think it was a problem, they just changed the way that they live their lives, because I find that with COPD patients, … They don't think they're any worse than they were a year before, but when you actually ask that question, they're quite considerably worse but they're not aware of it.

GP1: It's actually asking the question rather than waiting for them to present with a problem that they don't perceive as a problem.

GP3: And indeed, people who are retired, suddenly doing less functional activity, therefore they may not notice some of these symptoms. So obviously a lot of them don't go out of the house, or go out exercising or putting themselves under sort of pressure to do any activity which might bring out some of these symptoms. Whereas, if you're still at work or whatever, I suppose you might find tiredness and breathlessness might limit what you're doing during the day, and might be more of a functional problem that you'd come to see a doctor about. (FGD 2)

As the extract above also indicates, patients sometimes failed to recognise symptoms and unless GPs specifically asked about certain symptoms, such as voice changes, patients were unlikely to report them. In addition, GPs reported that smokers often experienced guilt for symptoms deemed “self‐inflicted”, and were consequently reluctant to report them to GPs. The reported duration and severity of symptomology by patients was often vague and reported differently between consultations (*the story changes*), further complicating diagnosis.

### 3.2 GP response to specific symptoms

NICE guidelines (NICE, 2005; NICE, 2015) recommend an urgent chest X‐ray (CXR) for patients with the following symptoms: unexplained haemoptysis; cough; fatigue; shortness of breath; chest pain, and; weight loss. The guidelines also recommend CXRs be offered to patients who present with recurrent chest infection. During FGDs, GPs were asked about two additional symptoms incorporated within IPCARD and potentially indicative of lung cancer: ongoing voice changes and non‐menopausal sweating (Brindle, Pope, Corner, Leydon, & Banerjee, 2012). GPs were asked to consider their experience of reviewing patients with these symptoms and the relative weight they gave to each symptom (see Table 3).

**Table 3:** GP perspectives on nine symptoms potentially indicative of lung cancer

Symptom	Views of GPs
Tiredness	Non‐specific symptom. Very common in general practice
	Tiredness alone is “almost never of significance”
	Perceived to be an “early” symptom
	Other symptoms experienced more acutely by patients if combined with tiredness
	Other symptoms are viewed more seriously if combined with tiredness
Cough	High prevalence expected due to of COPD amongst many ex‐/smokers population group
	Patients often think it is normal for smokers to have a cough
	Cough may last for 4–6 weeks post‐viral chest infection
	Only chronic cough (≥6 weeks) in the absence of recent infection would concern GPs
Breathing changes	Breathlessness a “fairly ubiquitous” symptom
	Progressively worse breathlessness a good indicative symptom of lung cancer
	Usually a late symptom, patients with lung cancer rarely present with it as a first symptom
	Older/inactive patients are less aware of their breathlessness/consider it normal
Sweats	Patients rarely present with sweats alone
	Important symptom when combined with cough
	An important symptom only if sweating is “profuse”/“drenching”
Chest infections	“Red flag” symptom if patient recently experienced many infections that do not settle
	A late sign of lung cancer
Unintentional weight loss	Always seen as a “red flag” symptom if sudden and significant
	Seen as a late symptom – “usually too late for survival”
	Diagnosis difficult if experienced as only symptom, as indicative of any tumour type
Chest pain	Patients subsequently diagnosed with lung cancer rarely present with chest pain
	Perceived as a “very late” symptom
	Often musco‐skeletal in origin, subsequent to coughing
Voice changes	Most common with laryngeal cancer
	Patients rarely present with this symptom
	GPs would refer anyone with dysphonia
Haemoptysis	Always a “red flag” symptom
	Refer for CXR immediately
	Seen as a “very late” symptom
	Sometimes caused by coughing

General practitioners considered many of these as “red flag” symptoms, but breathing changes, repeated chest infections, unintentional weight loss, chest pain and haemoptysis were all perceived as usually *late* symptoms when lung cancer is advanced. Breathing changes and tiredness were thought to be *fairly ubiquitous* of many illnesses, and tiredness, although a possible early sign of lung cancer, was thought to be *almost never of significance* on its own. However, GPs believed patients often experience other symptoms more severely when accompanied by tiredness, and GPs would tend to take other symptoms more seriously when accompanied by tiredness. Non‐menopausal sweats were identified as important only if they were what GPs indicated as *profuse* or *drenching* sweats. While participants reflected that patients rarely consulted for voice changes, they thought the symptom more common of laryngeal than lung cancer. Participants indicated that of the nine symptoms about which they were asked, those considered of most concern if the only ones present were haemoptysis, unintentional weight loss and persistent cough lasting longer than 6 weeks, although weight loss might be indicative of any tumour type. Other important signs identified during the FGDs included persistent hoarseness, and disturbed sleep caused by any of the other symptoms.

General practitioners described that while some patients were frequent attenders, others rarely if ever consulted GPs whatever symptoms they experienced. As the extract below illustrates, GPs in two FGDs argued that patients who rarely consulted the practice but then suddenly reported symptoms should trigger a high level of GP concern, irrespective of specific symptoms.

GP1: One of the biggest red flag symptoms for me when somebody comes to see me for anything are the people who never come to the surgery.

GP2: Absolutely.

GP1: Out of any of the symptoms, that is always more of a red flag to me than cough, breathlessness, and haemoptysis. If they hadn't been in for years and then even if it's something quite minor, I would investigate everything there and then. (FGD 3)

### 3.3 Investigating patients for lung cancer

Several GPs believed that in the absence of definitive symptoms presented by patients, their *gut instinct* was their *most valuable tool* when deciding to investigate for lung cancer. All GPs agreed they required a relatively low threshold of suspicion before referring patients with potential symptoms of lung cancer for CXRs. This would especially be the case for unexplained symptoms listed in the NICE guidelines, but might include others given the specific clinical history of a patient.

GP1: We've had it drummed into us, £20 for a chest X‐ray, then you get more radiation sat in a rock in Cornwall, so we're just told if in doubt, X‐ray.

GP2: And if in doubt repeat your X‐ray. That's been drummed into us again and again of course by the chest physicians, don't be afraid to do chest X‐rays, it's an easy test and it's cheap, low risk. (FGD 4)

Nevertheless, CXRs were perceived as *blunt instruments* and concern existed amongst GPs regarding the optimum timing of this investigation. Several participants were concerned that CXRs were often insufficiently sensitive to identify a lung tumour until quite large, and possibly inoperable. At the same time, as the GPs in the following extract argue, if CXRs were conducted when the mass was too small to be detected, then the negative result may serve as a false sense of security for both patient and GP.

GP1: And [patients] have not come back, because they've had a chest x‐ray and that's a false reassurance, it is about getting the time right.

GP2: I've had one in my career come back who we'd done a chest X‐ray fairly early on, and they came back about 6 months later with basically a consolidation on one side, and it had just completely obstructed. But they'd gone away because they'd had that reassuring chest X‐ray.

GP1: There is the danger of false reassurance, because you've had the symptom constellation, you've gone and had your chest X‐ray, the chest X‐ray's normal, the doctor told me my chest X‐ray's normal, I haven't got cancer, I won't go back if my cough continues, or actually I've coughed up a little bit of blood but I know my chest X‐ray was normal so. It is about the timing of that intervention, and acceptance that the chest X‐ray is not, …, it's far from a perfect test. (FGD 1)

Given these perceived limitations, participants from three FGDs argued GPs should have greater access to diagnostic tools such as computed tomography (CT). The GPs in the following extract argued that if they were to conduct a CT prior to referral to secondary care they would have greater confidence in their referral and it may also reduce time to diagnosis.

GP1: We often say to [patients], don't be put off if there's nothing on your first x‐ray, and to come back six weeks later if you're still worried and we'll do another one. That's again where I come back to that thing about access to CT scanning. By the time we're diagnosing them by CXR they're pretty big.

GP2: And the CXR misses quite a few.

GP1: Yes. The first thing the [specialist] respiratory team are going to do is [CT] scan them. If we had access we could do this and then only refer those that were really a concern. It would make the process clearer for us and cost the system less. (FGD 4)

Agreement about direct access to CT scanning was not universal, however, with some GPs indicating false negatives were similarly possible or that referring patients for CTs prior to secondary referral could slow rather than speed the process.

Despite the reportedly low threshold of suspicion to conduct a CXR, several GPs were less certain as to how they would proceed if the CXR result were inconclusive but symptoms persisted. As the following extract shows, a higher threshold of evidence appeared necessary for most GPs before referring patients to specialist secondary care teams, partly due to a concern that the *system would sink* if they referred all patients about whom they had low level suspicion.

GP1: It's a big step for us to then refer them on, and say, I know this chap's a smoker and he's got a persistent cough, his bloods are normal, his chest X‐ray shows nothing, I'd be grateful if you could exclude a lung cancer. We don't do many of those do we?

GP2: It's expected that we wouldn't, but if you went to a clinical meeting, that's not what the consultant exactly would say. But the system would sink in a week if we did that, it's just not what we do, but we'd have trouble defending that probably. So yes we'd be told, if the cough goes on, if the chest X‐ray's normal, then you probably should refer them on to us, but if we did that with them all, they don't realise the size of the problem in general practice.

GP3: I think there is a misconception isn't there, from [what] the hospitals think our workload is in respect to cough, persistent cough, and well, all the [lung cancer] symptoms. (FGD 4)

### 3.4 Potential for early diagnosis

Opinions differed amongst GPs with regards the potential for effectively increasing earlier diagnosis of lung cancer. While participants within all FGDs believed measures to identify symptoms of lung cancer at an early stage were necessary to offer patients greater opportunities for survival and quality of life, participants within two FGDs were pessimistic of their potential effectiveness. Moreover, while acknowledging the success of the national awareness “three‐week cough” campaign in 2012, almost all GPs expressed concerns regarding large perceived increases in patient consultations triggered by the campaign, over which practices had no control.

GP1: I quite like the idea of targeted screening. It appeals to me more than national campaigns because, again, national screening on the whole gets the worried well and just worries them more. Targeting those who ignore the national screening is a much better idea.

GP2: Particularly when you've got something like lung cancer …

GP1: But when you think about the amount of funding that must go into national campaigns. So if a slice of that came down to actually targeted screening, I imagine it could be so much more cost‐effective.

GP2: Something like that might work, but until you've tried it and crunched the numbers. But that would feel a bit more appropriate than this across the board [campaigns], because as [GP1 name] says, you can be guaranteed it's the worried well that come and snarl up the system. (FGD 5)

General practitioners considered symptoms indicative of lung cancer highly prevalent amongst older smokers/ex‐smokers, who often have associated comorbidities (ie, COPD, asthma). GPs argued viral chest infections often manifested with patients coughing for more than 3 weeks, and considered clinical histories, GP “hunches” and previous consulting behaviour as compelling as the presence of particular symptoms to identify suspected lung cancer. Consequently, participants were concerned that interventions should not simply target specific symptoms. Furthermore, participants favored practice‐level initiatives, where GPs and practice nurses identified patients with particular risk profiles, as potentially both as effective and cost effective as targeting single symptoms within the general population.

GP1: Thinking about my morning clinic, I was seeing patients with four of these [potential lung cancer] symptoms, but I haven't gone down the route of thinking lung cancer.

GP2: There is a risk we would generate workload that we wouldn't be able to cope with, and have a knock on risk of our other patients.

GP3: It's about stopping the flood gates opening.

GP1: It's not necessarily symptoms we should be targeting, anyway. It's particular patients. (FGD 3)

However, participants broadly agreed that whatever form targeted interventions took, *a core of people are not going to come whatever you do*.

## 4 Discussion

Limited work has previously investigated GP's views of their own role in early detection (Green, Atkin, & Macleod, 2015). This study conducted focus groups with GPs from participating general practice sites to elicit their views with regards facilitating targeted interventions. Participant GPs identified what they perceived as the three most relevant symptoms for diagnosing possible lung cancer as: recent, significant weight loss; persistent cough for longer than 6 weeks; and haemoptysis. There was no consensus between FGDs on those symptoms most indicative of *early* lung cancer. Previous research has found that lung cancer symptoms may be experienced only as vague or mild (Smith, Pope, & Botha, 2005), may be confounded by high levels of comorbidities (Stolper et al., 2011), and patients subsequently diagnosed with lung cancer reported good health prior to diagnosis (Brindle et al., 2012). Studies have shown patients with lung cancer commonly experience multiple and synchronous symptoms (Hamilton et al., 2005; Walter et al. 2015), and may be symptomatic for many months before presentation (Corner, Hopkinson, Fitzsimmons, Barclay, & Muers, 2005). Thus, patients are often unable to recognise all their symptoms (Smith et al., 2009), appraise symptoms as not warranting help‐seeking (Corner & Brindle, 2011), or else normalise symptoms, attributing their cause either to ageing processes or comorbidities (Corner et al., 2005; Tod & Joanne, 2010). Members of this research team have previously argued that to better elicit lung cancer symptoms, GPs may need to ask patients closed questions using non‐disease terminology (Brindle et al., 2012).

General practitioners emphasised that clinical histories and GP “hunches” were as important as specific symptoms in identifying patients who should be further investigated for lung cancer (Stolper et al., 2011). GPs also argued that symptomatic patients who rarely attended the practice, irrespective of their presenting symptoms, would trigger their concern. The CAPER studies and QCancer algorithms have provided risk prediction models for cancer types, including lung cancer (Hamilton, 2009; Hippisley‐Cox & Coupland, 2011). However, of a sample of patients who subsequently developed lung cancer, between 17% and 34% of symptoms presented in the previous 24 months were not caused by the cancer (Biswas, Ades, & Hamilton, 2015). Also, while hemoptysis is the strongest symptom predictor of lung cancer, only a fifth of patients experience it (Walter et al., 2015).

In response to symptoms, GPs reported they required low thresholds of suspicion of lung cancer before referring patients for CXRs; the standard initial investigation for symptoms indicative of lung cancer (NICE, 2005; NICE, 2011; NICE, 2015). Nevertheless, GPs expressed limited confidence in the diagnostic capacity of CXRs, and previous studies found few CXRs identified signs of lung cancer in patients 6 months or more prior to their actual diagnosis (Hamilton et al., 2005; Stapley, Sharp, & Hamilton, 2006). Uncertainty also existed amongst GPs regarding both how best to proceed if a patient's symptoms persist despite a negative CXR, and the level of suspicion appropriate before specialist respiratory referral should be made. The recently revised NICE guidance for suspected cancer referrals has reduced the expected probability threshold for a cancer diagnosis to trigger a secondary referral, from the previous predictive and prognostic value (PPV) threshold of approximately 5%–3% (NICE, 2015). Effectively, this means an extension in the number of patients needed to be referred (NNtR) for one cancer diagnosis from approximately 20 to 33. Nevertheless, GPs felt pressure not to refer patients with vague symptoms on to specialist secondary care without sufficient diagnostic evidence, and cited concern for “system overload”. It is therefore unclear how GPs will respond to a lower PPV threshold for cancer referrals and whether the guideline change will result in any meaningful behaviour change, hence referrals might remain restricted at practice level. Further research should explore whether and how GPs utilise the reduced PPV threshold. Although some GPs were ambivalent about greater access to additional diagnostic procedures such as CT, others argued it would give them greater confidence in the PPV of suspected lung cancer cases, better facilitating timely diagnosis. Investigating this potential is another area for future work.

While acknowledging the impact of the “3‐week cough” campaign, GPs argued such national initiatives often heightened demand on limited resources, but may have little impact upon those patients most at risk who ignore them. The existence of a symptomatic, non‐consulting group of primary care patients identified by our wider study would support this view (Wagland et al., 2016). Although the “3‐week cough campaign” led to a 9% increase in lung cancer diagnoses compared with the same period in the previous year (Ironmonger et al., 2015), there was a corresponding increase of >200,000 additional GP attendances and 30% increase in 2‐week waits recorded (PULSE 2014). Evidence also indicates such increases are particularly apparent in affluent rather than deprived areas (Green et al., 2015). All GPs in our sample recognised the importance for seeking methods for timely diagnoses of lung cancer, despite concerns about increased workloads. GPs within all FGDs agreed interventions targeting patients at high‐risk of lung cancer and who rarely attend primary care, might be at least as effective and cost‐effective as targeting specific symptoms. Potential methods, given this study's findings, would include practice‐level interventions that allowed GPs control over identifying and contacting “high‐risk” patients, facilitating planning for subsequent workload increases. Further work is needed to identify profiles of primary care patients who would benefit most from such targeted interventions.

### 4.1 Strength and limitations

Participant GPs had a broad range of experience (mean: 19 practice years), represented practices of different sizes, with both high and low levels of social deprivation, in both rural and urban settings, and consensus existed across FGDs on most themes.

## 5 Conclusion

General practitioners questioned current approaches to promoting earlier diagnosis through national campaigns. Given the problematic “symptom signature” and corresponding difficulties for both GPs and patients to recognise symptoms of lung cancer, future interventions promoting early diagnosis of lung cancer should include the targeting of “high‐risk” individuals. Some GPs also argued for greater access to more sensitive diagnostic investigations, in particular CT scans, to enhance the PPV of secondary referrals. Allowing GPs to target “at‐risk” patients on their lists would allow them to plan for the more limited workload increases these would entail compared with large national symptom campaigns over which they have limited control. Further work is required to identify primary care patients who would most benefit from such targeted interventions.
